# Predicting hypoglycemia in elderly inpatients with type 2 diabetes: the ADOCHBIU model

**DOI:** 10.3389/fendo.2024.1366184

**Published:** 2024-11-14

**Authors:** Rui-Ting Zhang, Yu Liu, Chao Sun, Quan-Ying Wu, Hong Guo, Gong-Ming Wang, Ke-Ke Lin, Jing Wang, Xiao-Yan Bai

**Affiliations:** ^1^ School of Nursing, Beijing University of Chinese Medicine, Beijing, China; ^2^ Nursing Department, Beijing Hospital, Beijing, China

**Keywords:** type 2 diabetes, hypoglycemia, logistic model, prediction, nomogram

## Abstract

**Background:**

Hypoglycemic episodes cause varying degrees of damage in the functional system of elderly inpatients with type 2 diabetes mellitus (T2DM). The purpose of the study is to construct a nomogram prediction model for the risk of hypoglycemia in elderly inpatients with T2DM and to evaluate the predictive performance of the model.

**Methods:**

From August 2022 to April 2023, 546 elderly inpatients with T2DM were recruited in seven tertiary-level general hospitals in Beijing and Inner Mongolia province, China. Medical history and clinical data of the inpatients were collected with a self-designed questionnaire, with follow up on the occurrence of hypoglycemia within one week. Factors related to the occurrence of hypoglycemia were screened using regularized logistic analysis(r-LR), and a nomogram prediction visual model of hypoglycemia was constructed. AUROC, Hosmer-Lemeshow, and DCA were used to analyze the prediction performance of the model.

**Results:**

The incidence of hypoglycemia of elderly inpatients with T2DM was 41.21% (225/546). The risk prediction model included 8 predictors as follows(named ADOCHBIU): duration of diabetes (*OR*=2.276, 95%*CI* 2.097˜2.469), urinary microalbumin(*OR*=0.864, 95%*CI* 0.798˜0.935), oral hypoglycemic agents (*OR*=1.345, 95%*CI* 1.243˜1.452), cognitive impairment (*OR*=1.226, 95%*CI* 1.178˜1.276), insulin usage (*OR*=1.002, 95%*CI* 0.948˜1.060), hypertension (*OR*=1.113, 95%*CI* 1.103˜1.124), blood glucose monitoring (*OR*=1.909, 95%*CI* 1.791˜2.036), and abdominal circumference (*OR*=2.998, 95%*CI* 2.972˜3.024). The AUROC of the prediction model was 0.871, with sensitivity of 0.889 and specificity of 0.737, which indicated that the nomogram model has good discrimination. The Hosmer-Lemeshow was *χ* ^2^ = 2.147 (*P*=0.75), which meant that the prediction model is well calibrated. DCA curve is consistently higher than all the positive line and all the negative line, which indicated that the nomogram prediction model has good clinical utility.

**Conclusions:**

The nomogram hypoglycemia prediction model constructed in this study had good prediction effect. It is used for early detection of high-risk individuals with hypoglycemia in elderly inpatients with T2DM, so as to take targeted measures to prevent hypoglycemia.

**Trial registration:**

ChiCTR2200062277. Registered on 31 July 2022.

## 1 Background

Hypoglycemia is a common complication in older adults with type 2 diabetes mellitus (T2DM) (1). Compared to the incidence of hypoglycemia in older adults with T2DM in the community (13.8%˜31.2%) (2–4), the incidence of hypoglycemia in hospitalized older adults with T2DM is higher, reaching 49% (5). Medication usage, dietary restrictions, irregular eating patterns, overtreatment, and the patient’s medical condition increase the risk of hypoglycemia in hospitalized older adults with T2DM (6, 7). Considering that the symptoms of hypoglycemia in older adults with T2DM may overlap with the primary diseases, the actual incidence of hypoglycemia may be higher. Hypoglycemic episodes cause varying degrees of damage in the functional system of elderly inpatients with T2DM, such as disorientation, difficulty concentrating, and impaired judgment, ultimately leading to dementia (8, 9). Hypoglycemia also increases hospitalization time and mortality in elderly inpatients (10). Compared with hyperglycemia, hypoglycemia is characterized by more urgent and dangerous onset, leaving a shorter reaction time for medical staff (11), and the treatment after its occurrence is also relatively delayed.

Early identification of the influencing factors of hypoglycemia and accurate risk assessment/prediction are of great significance for timely and effective hypoglycemia prevention. At present, the risk assessment of hypoglycemia is roughly divided into three methods: questionnaires based on clinical experience, based on physiological models and based on data mining technology. Using questionnaires based on clinical experience to assess the risk of hypoglycemia is an early research method, and there are few studies in this field. Accordingly, there are few questionnaires that can be used to assess the risk of hypoglycemia (12, 13). The risk prediction factors for hypoglycemia are influenced by multiple factors and their impacts on hypoglycemia vary, with some factors in constantly changing states (such as dietary, exercise, etc.). Using the questionnaires on clinical experience to predict the risk of hypoglycemia has low efficiency and poor prediction accuracy because the content lacks evidence supports, and each item has no weight. The risk prediction of hypoglycemia based on a physiological model is achieved by establishing equations based on pathological/physiological fundamental laws. Physiological models are mostly established based on patients’ blood sugar data (14, 15), but only focusing on blood sugar changes makes it difficult to comprehensively assess the risk of hypoglycemia. In addition, a continuous glucose monitoring system is not affordable for everyone, so the promotion and applicability of this method are greatly limited.

Predicting the risk of illness based on data mining technology has gradually grown in recent years. Data mining is defined as knowledge discovery in databases, and the commonly used methods include logistic regression (16). As a new information processing technology, data mining provides a new approach for predicting risk of hypoglycemia. The risk before hypoglycemia in diabetic patients and the hypoglycemia development process can be analyzed through data mining, such as analyzing the amount of exercise and dietary practices before hypoglycemia occurred. With the help of data mining, the advantage of Big Data in discovering signals can be fully utilized, providing effective methods and a basis for the early accurate identification of hypoglycemia signals.

There are few studies on hypoglycemia risk prediction of hypoglycemia in elderly inpatients with T2DM. The previous studies were single-factor prediction (such as only based on HbA1c) (17). Considering the complexity of influencing factors in hypoglycemia occurrence, multi-factor prediction is needed. So, this study screened factors related to hypoglycemia based on literature review and collected hypoglycemia related information of 546 hospitalized elderly patients, then developed a nomogram prediction model which was based on the findings of multi-factor regression analysis, integrating multiple predictive factors, and then transforming complex regression equations into visual graphs to forecast the probability of hypoglycemia in elderly inpatients with T2DM. The nomogram model simplifies the interpretation of predicted results and facilitates the assessment of the patient’s condition. The nomogram quantitative model of elderly inpatients with T2DM constructed in the study can be used in predicting the risk of hypoglycemia occurrence intuitively and conveniently, early identifying high-risk individuals of hypoglycemia, so as to take targeted intervention measures to improve blood glucose control of the patients.

## 2 Methods

This prediction model study is reported in accordance with the TRIPOD checklist (18). The STROBE checklist was used to guide the submission (19).

### 2.1 Participants

From August 2022 to April 2023, 546 older adults with T2DM were recruited by convenience sampling in the inpatient departments of seven tertiary-level general hospitals in Beijing and Inner Mongolia province, China.

Inclusion criteria: ① being diagnosed with type 2 diabetes (20); ② being in hospital; ③ ≥60 years old; ④ clear consciousness, no intellectual disability. Exclusion criteria: ① received hemodialysis or peritoneal dialysis in the past month.; ② combined with other serious diseases, such as malignant tumor; ③ language communication barriers; ④ moderate or severe cognitive impairment.

According to the principle of events per variable (21), 5˜10 patients for each independent variable are needed in logistic analysis. Therefore, the sample size required for this study is set to be 5 times the number of variables in the questionnaire (53 variables in this study), and taking a 10% loss of follow-up into account, the sample size needed for this study is 294 cases at least. The study was approved by the Ethics Committee of XXXXX (blinded for peer review).

### 2.2 Developed a questionnaire on related factors of hypoglycemia in elderly inpatients with T2DM.

#### 2.2.1 Screening the factors related to hypoglycemia in patients with T2DM

Factors related to hypoglycemia in patients with T2DM were included through literature review. Three subject terms– “type 2 diabetes,” “hypoglycemia/glucopenia/glucopenia” and “influencing factor/related factor/prediction factor/predictor “ –were used to search seven English databases including PubMed, Cochrane Library, JBI, EMBASE, Wiley Online Library, Web of Science, and ProQuest Database, and four Chinese databases including the Chinese Journal Full-text Database (CJFD), Wan Fang Database, VIP Chinese Science and Technology Journal Full-text Database, and Chinese Biomedical Literature Database.

A total of 5852 pieces of literature were retrieved, and the literature was screened according to the content. Finally, 760 articles were included in the study. After selecting the articles to use, the researchers selected corresponding quality evaluation tools based on the research type for literature quality appraisal, then extracted factors related to the risk of hypoglycemia from the 172 high-quality articles to develop the “Questionnaire on the factors related to hypoglycemia in elderly inpatients with T2DM”. The questionnaire included three parts, with a total of 53 factors: ① demographic data: gender, age, educational background, BMI and abdominal circumference; ② disease factors: duration of diabetes, history of hypoglycemia, diabetes drugs (oral hypoglycemic agents, insulin usage, oral hypoglycemic agents+ insulin), medication compliance, treatment plan adjustment, complications or comorbidity (13 items such as hypertension), and biochemical indicators (13 items such as urinary microalbumin); ③ lifestyle factors: drinking (drinking alcohol on an empty stomach, excessive drinking), smoking, diabetes education, diet (limiting the amount of carbohydrates, eating less, not eating on time), exercise (exercise time, intensity, frequency, and exercise on an empty stomach), blood glucose monitoring, malnutrition, depression symptoms, and cognitive impairment. The patients’ medical case records were consulted to determine whether patients had complications, comorbidity, and malnutrition.

Depression symptoms were measured with Patient Health Questionnaire-9 (PHQ-9). PHQ-9 was developed by Spitzer et al. (22) and is used to assess the frequency of depressive symptoms experienced by patients in the past two weeks. The scale consists of nine items, with a total score ranging from 0 to 27. A score of 0˜4 indicates no depression, 5˜9 indicates mild depression, 10˜14 indicates moderate depression, 15˜19 indicates moderately severe depression, and 20 indicates severe depression. The Cronbach’s α of PHQ-9 is 0.89, and the validity is 0.86 (23).

Cognitive impairment was confirmed with the Mini-Mental State Examination (MMSE). MMSE was developed by Folstein et al. (24) and introduced, translated, and revised in Chinese by Li Ge et al. (25). It is used to screen for cognitive function in older adults. MMSE includes four dimensions: orientation, attention and calculation ability, memory ability, and language ability, for a total of 30 items. The total score ranges from 0 to 30. The higher the score, the better the cognitive function. A total score ≤24 indicates cognitive impairment; 18˜24 indicates mild cognitive impairment; 16˜17 indicates moderate cognitive impairment; and 15 or less indicates severe cognitive impairment. The Chinese version of the scale has a reliability coefficient of 0.97 (26), and its validity is acceptable (27).

#### 2.2.2 Develop questionnaire with Delphi method

Delphi method was used to integrate expert opinions on feasibility and verbal expression of the items in the questionnaire formed in the previous stage. Moreover, according to expert opinions, the questionnaire added various biochemical test methods of the biochemical indicators and their corresponding time limit. Fifty-three factors identified in the previous stage were all retained, resulting in the final version of the “Questionnaire on the factors related to hypoglycemia in elderly inpatients with T2DM”.

### 2.3 Follow up on elderly inpatient’s blood glucose one week later

One week after the questionnaire was completed by the elderly inpatients with T2DM, the researcher followed up on the occurrence of hypoglycemia in the patients. According to standards issued by the American Diabetes Association (19), blood glucose < 70mg/dl (3.9mmol/L) is regarded as suffering hypoglycemia in this study.

### 2.4 Data collection

The study recruited older adults with T2DM in the participating hospitals’ inpatient departments, and the elderly inpatients were asked to complete a questionnaire within 1˜2 days of admission. The researchers used the MMSE to screen the cognitive function of the older adults at first. Elderly inpatients with normal cognitive function completed the questionnaire by themselves. If they had difficulty in filling it out, the researcher read questions and asked the patient to answer them. Patients with mild cognitive impairment were assisted by their main caregivers to complete the questionnaire. The researcher checked the questionnaire after it was completed. If there were any omissions or logic errors in the questionnaire, the researcher assisted the patient in filling it out or verifying the information again. The content of biochemical indicators was filled in by the researcher by checking the patient’s medical records. If there were missing items in a questionnaire, or there was a clear pattern in filling out the questionnaire items (such as all the item options being the same), or the filling of questionnaire options was invalid (such as garbled code), it was deemed as an invalid questionnaire. One week later, the researcher obtained information of whether the elderly inpatients had experienced hypoglycemia by asking the inpatient and reviewing their medical records in the hospital information system.

### 2.5 Statistical analysis

RapidMiner for Windows was used for the statistical analysis (28). The continuous variables were presented as mean ± SD, whereas the categorical variables were presented as number and percentage. First, the multiple collinearities of the regression equation were checked, and then regularized logistic (r-LR) analysis was performed to screen for predictive factors of hypoglycemia in elderly inpatients with T2DM. Next, a nomogram hypoglycemia prediction model was constructed. The area under the receiving operator curve (AUROC) was used to evaluate the discrimination ability of the prediction model. Sensitivity and specificity were calculated to verify the actual application performance of the prediction model. The Hosmer-Lemeshow goodness-of-fit test was used to assess the calibration of the prediction model. Decision Curve Analysis (DCA) was used to evaluate the clinical utility of the prediction model.

## 3 Results

### 3.1 Hypoglycemia incidence rate

A total of 561 elderly inpatients with T2DM were recruited, and all 561 completed the questionnaires. After excluding 15 invalid questionnaires, 546 samples were finally included. Participants were 60˜89 years old, with a hospital stay of 8˜15 days, averaging 11.48 ± 2.33 days (**Table 1**). Among them, 225 experienced hypoglycemia (blood glucose 2.1~3.9 mmol/L) within one week after filling out the questionnaire, and the incidence of hypoglycemia was 41.21%.

**Table 1:** Comparison of general information of elderly inpatients with T2DM.

Characteristics		Total (*n*=546)	Group		
			Hypoglycemia group (*n*=225)	Non-hypoglycemia group (*n*=321)	*t*/*χ^2^*
Age (years old)		76.02 ± 5.62	78.06 ± 3.59	73.35 ± 8.72	2.549^*^
BMI(kg/m^2^)		25.31 ± 5.19	24.69 ± 3.19	23.30 ± 3.43	1.618
Abdominal circumference (cm)		86.49 ± 5.88	83.64 ± 5.52	85.53 ± 5.72	2.567
Duration of diabetes (years)		22.89 ± 12.63	24.10 ± 12.58	22.02 ± 12.29	3.442^*^
Gender	Male	306	126	180	1.233
	Female	240	99	141	
Educational background	Primary school and below	133	62	71	1.075
	Middle school	266	102	164	
	College and above	147	61	86	
Hypertension	Yes	339	160	179	3.824^**^
	No	207	65	142	
Cognitive impairment	Mild	163	93	70	1.684^*^
	Moderate	23	13	10	
	No	360	119	241	
Depression symptoms	Yes	65	14	51	1.195
	No	481	211	270	
Diabetes treatment	Oral hypoglycemic agent	192	85	107	2.559
	Insulin	317	122	195	
	Oral hypoglycemic agents and insulin	37	18	19	
Adjustment of hypoglycemic program	Yes	248	161	87	4.627*
	No	298	64	234	

*P<0.05; **P<0.01.

### 3.2 Multivariate analysis

Regularized logistic analysis was performed with the occurrence of hypoglycemia as the dependent variable and 53 factors mentioned above were taken as the independent variables. There is no multicollinearity among independent variables (*VIF*=1.6).

The risk prediction model included eight predictors as follows: duration of diabetes, urinary microalbumin, oral hypoglycemic agents, mild cognitive impairment, insulin usage, hypertension, blood glucose monitoring, and abdominal circumference. OR value of abdominal circumference is the largest (**Table 2**).

**Table 2:** Logistic regression analysis of hypoglycemia in elderly inpatients with T2DM.

Variable	*β*	SE	Wald *χ* ^2^	*P-*value	Odds ratio	95%*CI*
Duration of diabetes	1.104	0.623	5.88	0.002	2.276	2.097˜2.469
Urinary microalbumin	1.006	0.415	0.14	<0.001	0.864	0.798˜0.935
Oral hypoglycemic agents	0.832	0.680	3.32	0.020	1.345	1.243˜1.452
Cognitive impairment	0.764	0.344	2.84	0.041	1.226	1.178˜1.276
Insulin usage	0.477	0.152	5.41	0.007	1.002	0.948˜1.060
Hypertension	0.433	0.095	1.97	0.039	1.113	1.103˜1.124
Blood-glucose monitoring	-0.405	0.434	2.65	0.014	1.909	1.791˜2.036
Abdominal circumference	-1.903	0.680	1.43	<0.001	2.998	2.972˜3.024

The regression equation of the model was obtained as follows: Logit(P)= 1.104×duration of diabetes +1.006×urinary microalbumin +0.832×oral hypoglycemic agents +0.764×mild cognitive impairment +0.477×insulin usage+0.433×hypertension -0.405×blood glucose monitoring -1.903×abdominal circumference -0.435.

### 3.3 Nomogram prediction model construction

**Figure 1:**
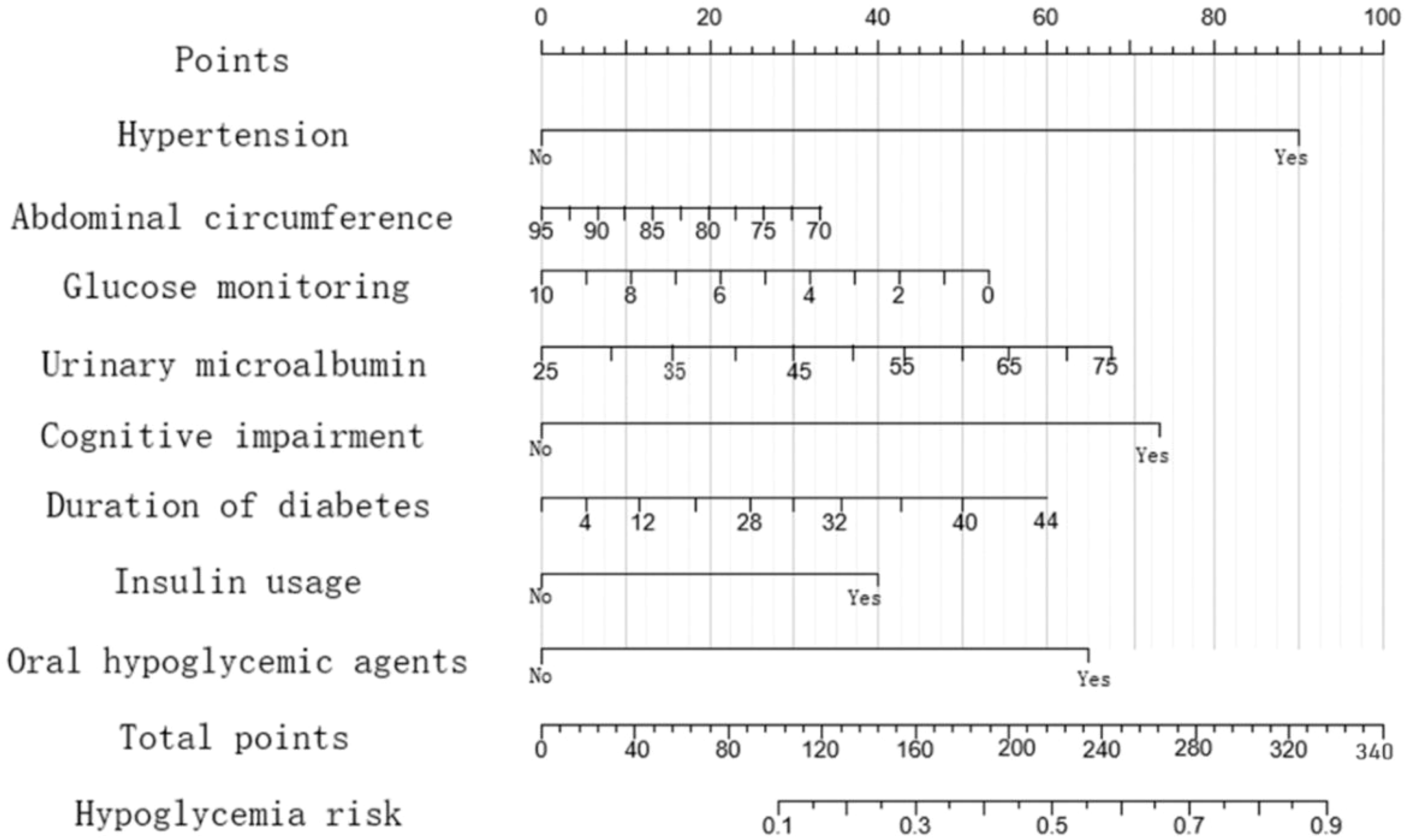
Nomogram hypoglycemia risk prediction model for elderly inpatients with T2DM.

The regression coefficients of the eight factors obtained from regularized logistic regression were used as the weights of the eight factors in the prediction model, and a nomogram hypoglycemia risk prediction model was established (**Figure 1**). The single score corresponding to each variable under different values is the score shown in **Figure 1** (the default is 0~100 points), and the score of the eight variables added together is the total score (“total points” in **Figure 1**). The line “Hypoglycemia risk” in **Figure 1** indicates the risk of hypoglycemia predicted by the multivariate model. The total score ranges from 0 to 340, and the corresponding “Hypoglycemia risk” ranges from 0.1 to 0.9. The higher the total score, the greater the risk of hypoglycemia. The practical application method is as follows: draw a vertical line upward at the position corresponding to the patient’s situation to get the corresponding single score, add the scores of all variables to get the total score (total points) of the patient, and then draw a vertical line downward from the “total points” to get the “predicted risk” of hypoglycemia of the patient.

### 3.4 Model effect evaluation

**Figure 2:**
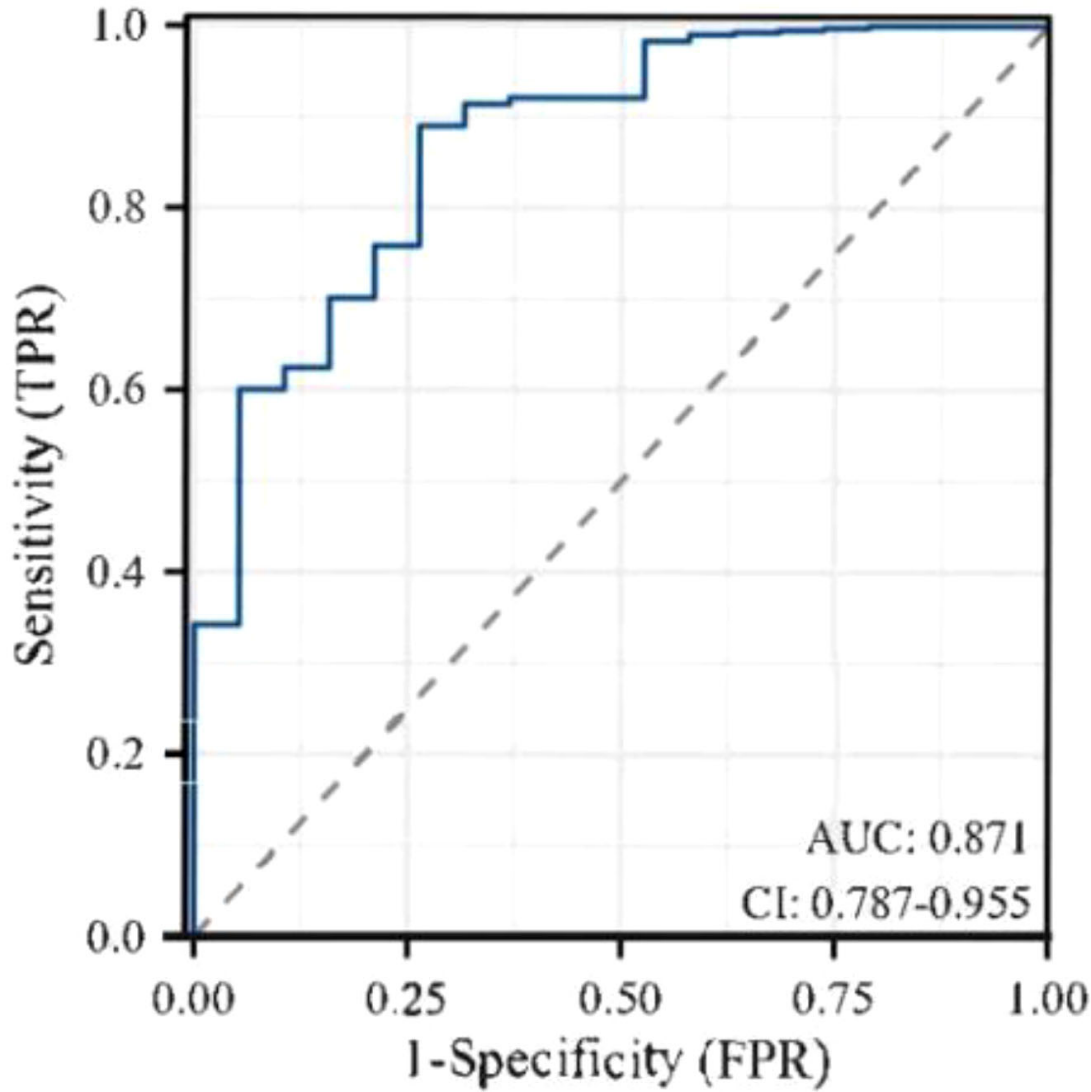
ROC curve of Nomogram hypoglycemia risk prediction model.

**Figure 3:**
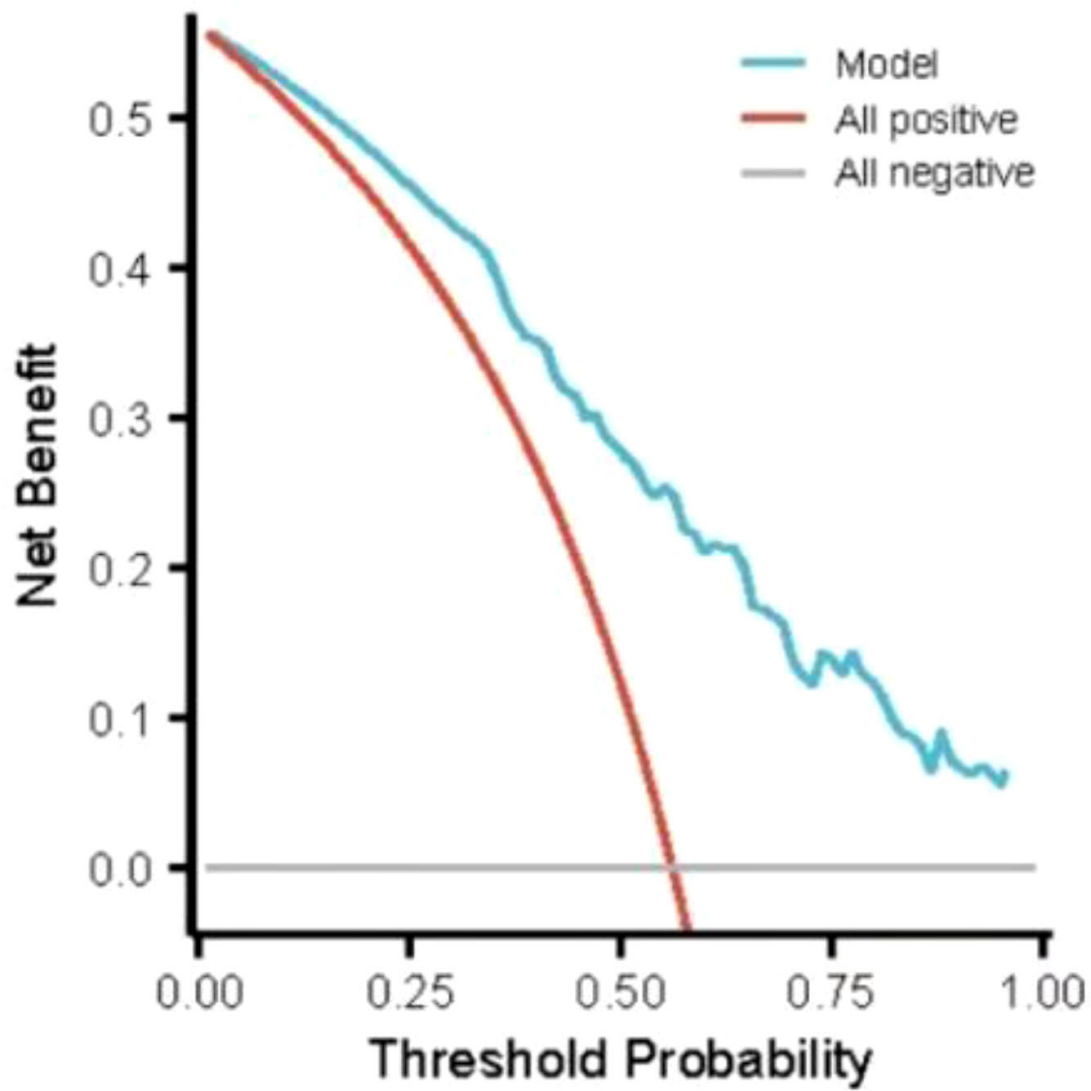
DCA curve of Nomogram hypoglycemia risk prediction model.

Through goodness-of-fit test analysis, Hosmer-Lemeshow *χ* ^2^ = 2.147, *P* = 0.75 (*P* > 0.05 indicated that the model had excellent goodness-of-fit) (29). The ROC curve was used to test the fitting effect between the patient’s model score and the patient’s actual hypoglycemia (**Figure 2**), and the AUROC was 0.871 (> 0.75), and the 95% CI was 0.787˜0.955, with sensitivity and specificity of 0.889 and 0.737, respectively. In the nomogram prediction model constructed in this study, DCA is higher than all positive lines and all negative lines in the range of x values 0~1 (horizontal axis), which shows that this model has good clinical applicability and good prediction ability, as shown in **Figure 3**.

## 4 Discussion

This study collected hypoglycemic related data from 546 hospitalized older adults with T2DM in seven hospitals in Beijing and Baotou, two cities in the north of China with similar climatic conditions and food structure systems, and constructed a nomogram model to predict the hypoglycemic risk of elderly inpatients with T2DM. The nomogram model transforms complex equations into visual images and uses numerical probabilities to represent the possibility of hypoglycemia occurrence, increase the readability of results, and facilitate individualized hypoglycemia risk assessment and early identification of high-risk patients, in order to guide patients to appropriately change their lifestyles or adjust therapeutic plans in time to prevent the occurrence of hypoglycemia.

In this study, regularized logistic regression was used to screen 53 potential factors. Finally, eight factors, including the abdominal circumference, duration of diabetes, blood glucose monitoring, oral hypoglycemic agents’ usage, cognitive impairment, hypertension, and insulin usage were included as predictive factors for hypoglycemia in elderly inpatients with T2DM. Patients’ information of the eight factors can be collected through medical records, inquiries, scale measurements and examinations, with high clinical operability and practicality.

Abdominal circumference was the most important predictor of hypoglycemia in elderly inpatients with T2DM, and the greater the abdominal circumference, the lower the risk of hypoglycemia. Patients with larger abdominal circumferences have excessive abdominal fat distribution, which can cause insulin resistance and high blood glucose levels (30). Although large abdominal circumference is harmful to physical health, a small abdominal circumference can increase the risk of hypoglycemia. A large-sample study on risk factors for hypoglycemia in patients with T2DM showed that, for every 3cm increases in abdominal circumference, the risk of hypoglycemia decreases by 7.1% (31). An RCT showed that for every 1 cm decrease in abdominal circumference, the risk of severe hypoglycemia in older adults with T2DM increased 7% (32). Duration of diabetes of the elderly inpatients with T2DM is positively correlated with the occurrence of hypoglycemia, which is consistent with the previous study (33). Yotsapon et al. (34) has demonstrated a significantly increased risk of hypoglycemia in elderly patients with T2DM whose duration of diabetes was ≥ 6 years.

The biochemical indicator “urinary microalbumin” was the predictor of hypoglycemia in elderly inpatients with T2DM in this study. The higher the urinary microalbumin value, the higher the risk of hypoglycemia, which is similar to the research results of Hodge et al. (35) and Nakhleh et al. (36). Urinary microalbumin is a routine examination item for diabetes patients during hospitalization. Urinary microalbumin above the normal level suggests that patients may have an early renal injury, which affects the excretion of insulin, leading to insulin accumulation in the patient’s body and susceptibility to hypoglycemia (37). With the widespread use of hypoglycemic drugs, hypoglycemia events are also on the rise (38). In the study, all of the elderly inpatients used hypoglycemic agents: 58.06% insulin, 35.16% used oral hypoglycemic agents, and 6.78% used both. Our study found that the use of oral hypoglycemic agents or insulin is a predictor of hypoglycemia, which is consistent with previous studies in which it was reported that hypoglycemia is commonly caused by insulin, sulfonylurea, and non-sulfonylurea insulin secretagogues (39, 40).

Cognitive impairment is also an essential factor in predicting hypoglycemia in elderly inpatients with T2DM, and cognitive impairment increases the risk of hypoglycemia. For older adults with T2DM, cognitive impairment can lead to difficulties in controlling blood glucose and identifying and treating hypoglycemia, and frequent hypoglycemia attacks can further exacerbate cognitive decline, forming a vicious cycle (41). In addition, hypertension is a predictor for hypoglycemia. Previous research has demonstrated that patients with concomitant hypertension have an increased risk of hypoglycemia (42–44). Moreover, the frequency of blood glucose monitoring is also a predictor of hypoglycemia in elderly inpatients with T2DM. The higher the frequency of glucose monitoring, the lower the risk of hypoglycemia, which is similar to the results of previous studies (45). Regular blood glucose monitoring helps to develop personalized treatment plans and enhances self-management capabilities, which improves blood glucose control in elderly inpatients with T2DM (46).

The ADOCHBIU nomogram hypoglycemia risk prediction model based on regularized logistic regression has a good predictive ability for hypoglycemia in elderly inpatients with T2DM. It helps medical staff identify older adults with T2DM in a high risk of hypoglycemia during hospitalization and take target interventions to prevent hypoglycemia effectively. There are some limitations in this study. Firstly, the elderly inpatients in this study came from northern China, so the applicability of risk prediction may be limited. To broaden the scope, we plan to recruit elderly inpatients with T2DM in cities in southern, western, and eastern China, which will optimize the prediction model. Secondly, although elderly inpatients in this study were regularly monitored for blood glucose, no dynamic blood glucose monitoring was carried out. Considering a small part of older adults may suffer from asymptomatic hypoglycemia [13% (47)], the incidence of hypoglycemia in this study may be low, and the nomogram model is not suitable for patients with asymptomatic hypoglycemia for the time being. If conditions permit, continuous glucose monitoring for the construction of such models is recommended.

## 5 Conclusions

The incidence of hypoglycemia among elderly inpatients with T2DM was 41.21%. The logistic regression equation included eight predictors(named ADOCHBIU): duration of diabetes, urinary microalbumin, oral hypoglycemic agents, mild cognitive impairment, insulin usage, hypertension, blood glucose monitoring, and abdominal circumference. The ADOCHBIU nomogram hypoglycemia risk prediction model for elderly inpatients with T2DM was constructed based on the logistic regression equation and had good predictive performance. In the ADOCHBIU nomogram hypoglycemia risk prediction model, the higher the total score, the greater the risk of hypoglycemia.

## Data Availability

The raw data supporting the conclusions of this article will be made available by the authors, without undue reservation.
